# RETRACTION: “Gastroprotective Properties of Karanjin from Karanja (*Pongamia pinnata*) Seeds; Role as Antioxidant and H^+^, K^+^‐ATPase Inhibitor”

**DOI:** 10.1155/ecam/9850704

**Published:** 2026-02-10

**Authors:** Evidence-Based Complementary and Alternative Medicine

RETRACTION: Vismaya, S. M. Belagihally, S. Rajashekhar, V. B. Jayaram, S. M. Dharmesh, and S. K. C. Thirumakudalu, “Gastroprotective Properties of Karanjin from Karanja (Pongamia pinnata) Seeds; Role as Antioxidant and H+, K^+^‐ATPase Inhibitor,” *Evidence-Based Complementary and Alternative Medicine*, no. 2011 (2011), https://doi.org/10.1093/ecam/neq027.

The above article, published online on 13 March 2011 in Wiley Online Library (https://wileyonlinelibrary.com), has been retracted by John Wiley & Sons Ltd.

The retraction has been agreed following an investigation of the concerns raised by *Penicillium citreoviride* and *Actinopolyspora biskrensis* on PubPeer [1], which identified several instances of similarity between the western blot bands in the following figures:

‐ Figure 2: The images of the Swim Stress and Oil + ES ulcers appear identical after a 90° rotation.

‐ Figure 4: Unexpected similarities have been identified between the images of the Omeprazole control and Omeprazole + ES groups. Additional concerns have been raised regarding the similar features of ethanol Stress and Oil + ES panels.

As a result of the investigation, the data and conclusions of this article are considered unreliable.

The authors have been informed of this decision but did not provide a response.
